# Aldosterone Excess Induced Mitochondria Decrease and Dysfunction via Mineralocorticoid Receptor and Oxidative Stress In Vitro and In Vivo

**DOI:** 10.3390/biomedicines9080946

**Published:** 2021-08-02

**Authors:** Cheng-Hsuan Tsai, Chien-Ting Pan, Yi-Yao Chang, Shih-Yuan Peng, Po-Chin Lee, Che-Wei Liao, Chia-Tung Shun, Po-Ting Li, Vin-Cent Wu, Chia-Hung Chou, I-Jung Tsai, Chi-Sheng Hung, Yen-Hung Lin

**Affiliations:** 1 Graduate Institute of Clinical Medicine, College of Medicine, National Taiwan University, Taipei 100, Taiwan; cheng.hsuan.richard.tsai@gmail.com (C.-H.T.); rollerpapa@gmail.com (Y.-Y.C.); 2 Department of Internal Medicine, National Taiwan University Hospital Jinshan Branch, New Taipei City 208, Taiwan; 3 Department of Internal Medicine, Division of Cardiology, College of Medicine, National Taiwan University Hospital, National Taiwan University, Taipei 100, Taiwan; pan.chienting.m@gmail.com (C.-T.P.); sypeng0302.12@gmail.com (S.-Y.P.); yo.ahliao@gmail.com (C.-W.L.); 101711140@gms.tcu.edu.tw (P.-T.L.); austinr34@gmail.com (Y.-H.L.); 4 Department of Internal Medicine, National Taiwan University Hospital Yun-Lin Branch, Yun-Lin 640, Taiwan; 5 Cardiology Division of Cardiovascular Medical Center, Far Eastern Memorial Hospital, New Taipei City 220, Taiwan; 6 Department of Medical Imaging, College of Medicine, National Taiwan University Hospital, National Taiwan University, Taipei 100, Taiwan; bochinglee@gmail.com; 7 Department of Medicine, National Taiwan University Cancer Center, Taipei 106, Taiwan; 8 Department of Forensic Medicine and Pathology, National Taiwan University Hospital, Taipei 100, Taiwan; ctshun@ntu.edu.tw; 9 Division of Nephrology, Department of Internal Medicine, College of Medicine, National Taiwan University Hospital, National Taiwan University, Taipei 100, Taiwan; dr.vincentwu@gmail.com; 10 Department of Obstetrics and Gynecology, College of Medicine, National Taiwan University Hospital, National Taiwan University, Taipei 100, Taiwan; ch640124@gmail.com; 11 Division of Nephrology, Department of Pediatrics, College of Medicine, National Taiwan University Hospital, National Taiwan University, Taipei 100, Taiwan; 12 Cardiovascular Center, National Taiwan University Hospital, Taipei 100, Taiwan

**Keywords:** aldosterone, mitochondrial dysfunction, primary aldosteronism, heart failure, oxidative stress

## Abstract

Aldosterone excess plays a major role in the progression of cardiac dysfunction and remodeling in clinical diseases such as primary aldosteronism and heart failure. However, the effect of aldosterone excess on cardiac mitochondria is unclear. In this study, we investigated the effect of aldosterone excess on cardiac mitochondrial dysfunction and its mechanisms in vitro and in vivo. We used H9c2 cardiomyocytes to investigate the effect and mechanism of aldosterone excess on cardiac mitochondria, and further investigated them in an aldosterone-infused ICR mice model. The results of the cell study showed that aldosterone excess decreased mitochondrial DNA, COX IV and SOD2 protein expressions, and mitochondria ATP production. These effects were abolished or attenuated by treatment with a mineralocorticoid receptor (MR) antagonist and antioxidant. With regard to the signal transduction pathway, aldosterone suppressed cardiac mitochondria through an MR/MAPK/p38/reactive oxygen species pathway. In the mouse model, aldosterone infusion decreased the amount of cardiac mitochondrial DNA and COX IV protein, and the effects were also attenuated by treatment with an MR antagonist and antioxidant. In conclusion, aldosterone excess induced a decrease in mitochondria and mitochondrial dysfunction via MRs and oxidative stress in vitro and in vivo.

## 1. Introduction

Aldosterone excess plays a major role in the progression of cardiac dysfunction and remodeling in clinical diseases such as heart failure and primary aldosteronism (PA). Activation of the renin–angiotensin–aldosterone system (RAAS) and increased aldosterone production has been shown to play a major role in the progression of heart failure, particularly in patients with systolic dysfunction [1,2]. Aldosterone promotes cardiomyocyte inflammation and participates in the progression of cardiac remodeling [3,4,5]. In addition, the excessive endogenous aldosterone production in PA, which is unresponsive to renin regulation induces cardiac remodeling including fibrosis and hypertrophy and it is associated with worse cardiovascular outcomes [3,6].

Mitochondria are double-membrane intracellular organelles involved in cellular energy generation, reactive oxygen species (ROS) regulation and apoptosis [7]. Mitochondria are especially important in cardiomyocytes which have high bioenergetic demands, and more than 90% of ATP is generated from mitochondria [8,9]. Mitochondria are not only a major source of ROS but also a major target for ROS damage. The accumulation of ROS is significantly enhanced in failing myocardium and is associated with mitochondrial dysfunction [10]. Previous studies have shown that aldosterone and mineralocorticoid receptor (MR) activation are also important sources of ROS generation [5]. In addition, mitochondrial dysfunction has been strongly associated with the development and further progression of cardiac remodeling and heart failure [11,12,13]. However, the impact of aldosterone on cardiac mitochondria and its molecular mechanisms are unclear. Therefore, the aim of this study was to investigate the effect of aldosterone excess on cardiac mitochondria and its mechanisms.

## 2. Materials and Methods

### 2.1. Preparation of Rat Ventricular H9c2 Cells, Mouse Primary Cardiomyocytes, and Human AC16 Cardiomyocytes

The rat embryonic cardiomyocyte H9c2 cell line was purchased from ATCC (ATCC, Manassas, VA, USA) and maintained in ATCC formulated Dulbecco’s modified Eagle’s medium (Catalog No. 30 2002) containing 10% fetal bovine serum (Gibco™ Fetal Bovine Serum, Waltham, MA, USA). The cells were cultured in a humidified atmosphere of 95% air and 5% CO_2_ at 37 °C.

Eight–week–old C57BL/6 male mice were euthanized by inhalation of CO2. This animal study protocol was approved by the Institutional Animal Care and Use Committee (IACUC) of the College of Medicine and College of Public Health, National Taiwan University. The hearts were dissected and kept in ice cold normal saline. The mouse primary cardiomyocytes were purified using the method described in [14]. Cells were cultured at 37 °C in an incubator containing 5% CO2 until use in experiments.

Human AC16 cardiomyocytes Cell Line (SCC109) were purchased from Sigma–Aldrich and cultured in Dulbecco’s modified Eagle’s medium (DMEM; Gibco) supplemented with 10% fetal bovine serum (FBS; Gibco) at 37 °C in an atmosphere containing 5% CO_2_.

### 2.2. Flow Cytometry for Annexin-V

The status of apoptosis of cardiomyocytes was determined by quantifying Annexin V/PI staining. With the FACS scanner and Cell Quest software (Becton Dickinson Immunocytometry Systems, San Jose, CA, USA) according to manufacturer’s instructions from the Alexa Fluor^®^ 488 Annexin V/Dead Cell Apoptosis Kit (Thermo Fisher Scientific Inc., Waltham, MA, USA).

### 2.3. Mitochondrial DNA Copy Number Determined by Quantitative Polymerase Chain Reaction (qPCR)

Total DNA from the H9c2 cells or mouse cardiac tissue was extracted using a DNeasy Blood & Tissue Kit (Qiagen, Valencia, CA, USA) according to the manufacturer’s instructions. Fifty nanograms of DNA was used for real–time qPCR. The relative mitochondrial DNA copy number was measured by qPCR with primer sets against mitochondrial DNA–encoded d–Loop: 5′–ATCCTCCGTGAAATCAACAA–3′ and 5′–CAGGACTTTGTGCTGACCTT–3′. This was corrected by simultaneous measurements of nuclear DNA with the β–tubulin gene using the following primers: forward, 5′–GTTTTGGGAGGTCATCAGTG–3′ and reverse, 5′–CCAGTTATTTCCTGCACCAC–3′ [15].

### 2.4. Reagents and Chemical Inhibitors

Aldosterone, eplerenone (an MR blocker), SB203580 (an MAPK/p38 inhibitor), PD98059 (an MEK/ERK inhibitor), LY294002 (a PI3K/ AKT inhibitor) and NAC (an antioxidant) were purchased from Sigma (St. Louis, MO, USA). Aldosterone, eplerenone and the other chemical inhibitors were dissolved in dimethyl sulfoxide (DMSO). NAC was dissolved in ddH2O (double–distilled water).

### 2.5. Immune Fluorescence Staining for Mitochondria COX IV Protein and Mitochondrial Dynamics Assessments

A coverslip was placed in a 6-cm culture dish onto which H9c2 cells, AC16 or primary mouse cardiomyocytes (1 × 10^5^ cells/well) were seeded. After experimental treatment, the H9c2 cells were fixed in 4% paraformaldehyde/phosphate-buffered saline (PBS) for 15 min. The cells were then blocked with 5% non-fat milk/PBS for 30 min at room temperature. The slides were incubated with anti-mitochondrial cytochrome c oxidase (COX) subunits, complex IV (COX IV) antibody (sc–58348, Santa Cruz Biotechnology, Inc.; Santa Cruz, CA, USA), anti-dynamin-related protein 1 (Drp1) antibody (sc–271583, Santa Cruz Biotechnology, Inc.) or anti-mitofusin-2 (Mfn2) antibody (sc–515647, Santa Cruz Biotechnology, Inc.) for 16 h at 4°C. The fluorescence-conjugated secondary antibody was incubated for 1 h at room temperature. The slides were then washed three times with PBS containing 1% bovine serum albumin (BSA), incubated with 4′,6–diamidino–2–phenylindole (DAPI) (Sigma; St. Louis, MO, USA) for 30 min, washed a further three times with PBS containing 1% BSA, wet-mounted in Prolong™ antifade (Molecular Probes Inc., Eugene, OR, USA), and sealed with nail varnish. Cells were photographed under a fluorescence microscope. On the other hand, cardiomyocytes were also seeded into a 96-well plate (1 × 104 cells/well) for treatment. Intensity of fluorescence was measured using a Beckman Coulter DTX 880 Multimode Detector.

### 2.6. Enzyme-Linked Immunosorbent Assay (ELISA)

The superoxide dismutase 2 (SOD2) level in cell lysates was determined using a SOD2 DuoSet IC ELISA kit (DYC3419–2; R&D Systems, Minneapolis, MN, USA). The cytosolic cytochrome c in cell lysates was determined using a Rat/Mouse Cytochrome c Quantikine ELISA Kit (MCTC0; R&D Systems, Minneapolis, MN, USA). Cytosolic protein was purified after experimental treatment according to the manufacturer′s instructions. The concentration of cytosolic proteins was quantified using a Bio-Rad protein assay reagent (Bio-Rad Laboratories, Hercules, CA, USA).

### 2.7. Detection of ATP Production

The level of H9c2 cellular ATP was determined using a Seahorse Extracellular Flux Analyzer via a Luminescent ATP Detection Assay Kit (North Billerica, MA, USA), according to the manufacturer’s instructions.

### 2.8. Western Blot for Mitochondria, Cytosolic and Mitochondrial Dynamics Related Protein Assessments

The total proteins of H9c2 cells or cardiac ventricle tissue (homogenization of tissues by grinding on liquid nitrogen) were extracted using a total protein extraction kit (Merck Millipore, Billerica, MA, USA), and the protein concentrations were measured using a Bio-Rad protein assay. Protein samples (30 µg) were separated using 10% SDS–PAGE (sodium dodecyl sulfate polyacrylamide gel electrophoresis) and then transferred onto PVDF (polyvinylidene difluoride, Merck Millipore Durapore™) membranes. The membranes were blocked with 5% non-fat milk for the non-phosphorylated form of protein, or with 5% BSA for the phosphorylated form of protein for 30 min. The membranes were then immunoblotted with the following primary antibodies for 1 h at room temperature: glyceraldehyde–3–phosphate dehydrogenase (GAPDH), α–Tubulin, COX IV, Drp1 and Mfn2 antibodies (Santa Cruz Biotechnology, Santa Cruz, CA, USA). The bound antibodies on the membranes were detected using peroxidase-coupled secondary antibodies for 30 min. Signals were detected by adding commercial chemiluminescent detection reagents (Thermo Fisher Scientific, Waltham, MA, USA). A digital imaging system (Bio Pioneer Tech Co., New Taipei City, Taiwan) was used to detect the signals, which were further analyzed using ImageJ^®^ software. Furthermore, the membranes on which COX IV, Drp1 or Mfn2 were first detected as described above were stripped, and levels of GAPDH protein were determined. Membrane stripping was carried out by incubating the membranes in stripping buffer (37.5 mM Tris, pH 6.8, 2% SDS, 1% β–mercaptoethanol at 56 °C for 20 min. The stripped membranes were washed three times with PBS with Tween (10 mM Tris, pH 7.5, 150 mM NaCl, 0.05% Tween 20).

### 2.9. Drp1 and Mfn2 Real-Time Quantitative RT-PCR

Total RNA was isolated from cardiomyocytes using a RNAzol B reagent (Biotecx Laboratories, Houston, TX, USA) according to the manufacturer’s instructions, and then cDNA was prepared from 2 μg of the total RNA with random hexamer primers according to the cDNA synthesis ImProm–II protocol (Promega, Madison, WI, USA). The specific oligonucleotide primer pairs for mouse Drp1: forward primer ATGCCAGCAAGTCCACAGAA, reverse primer TGTTCTCGGGCAGACAGTTT. Mfn2: forward primer TGCACCGCCATATAGAGGAAG, reverse primer TCTGCAGTGAACTGGCAATG, Beta–actin forward primer AGAAGCTGTGCTATGTTGCTCTA, reverse primer TCAGGCAGCTCATAGCTCTTC [16]. The cycle threshold (CT) values of all candidate genes were normalized to β–actin as a reference gene, and the ΔCT values were calculated. The results were plotted as fold changes relative to the vehicle group.

### 2.10. ROS Detection

ROS formation in the H9c2 cells was determined using the fluorescent dye 2′,7′–dichlorofluorescin diacetate (DCF–DA, Sigma). Two days before the experiments, H9c2 cells were seeded into a six-well plate (5 × 10^4^ cells/well). The cells were incubated with DCF-DA (5 mM) for 30 min at 37 °C and then washed with PBS prior to incubation at the experimental conditions. ROS accumulation was measured using a fluorescence microscope. The DCF–DA fluorescent emission was measured using a Beckman Coulter DTX 880 Multimode Detector at a wavelength of 530 nm, with excitation at a wavelength of 488 nm.

### 2.11. Animal Model

Eight-week-old ICR male mice were purchased from the Animal Center of the Medical College of National Taiwan University and kept in standard animal housing conditions. The protocol of this experiment was approved by the Animal Care and Use Committee of the Medical College of National Taiwan University. All animal experiments conformed to the NIH guidelines (Guide for the Care and Use of Laboratory Animals). Mice with a similar body weight (25–30 g) were used in the treatment groups (vehicle, aldosterone, aldosterone with eplerenone (50 mg/kg/day), aldosterone with NAC (1000 mg/kg/day) and aldosterone with PBS as vehicle.

In the aldosterone treatment group, the mice were processed with right nephrectomy. After 1 week, a 21-day continuous aldosterone release pellet (Innovative Research of America, Sarasota, FL, USA, 0.25 mg/pellet, 0.11 mg/day) was implanted subcutaneously, and 1% sodium chloride was given in the drinking water. In the control group, a placebo pellet (vehicle only) was implanted. For the continuous aldosterone infusion model, the pellets were changed every 20 days. During implantation of the pellets, the mice were anesthetized with inhaled 5% isoflurane. The mice were sacrificed after 2, 4 or 6 weeks by carbon dioxide inhalation. Briefly, the mice were placed into a chamber without pre-charging. A fill rate of about 10% to 30% of the chamber volume per minute with 100% CO_2_ was used, and the mice were unconscious within 2 to 3 min. We maintained the CO_2_ flow for a minimum of 1 min after respiration had ceased. Then we removed the mice from the chamber and performed the experiments. The heart tissue was dissected; part of the ventricles tissue was processed for immunohistochemical staining, part of the ventricles tissue was processed for western blotting as described above.

### 2.12. Immunohistochemical Staining of Mouse Myocardium

Mouse heart tissue sections were deparaffinized, rehydrated, and microwaved in 0.01 M citrate buffer (pH 6.0) for antigen retrieval. The sections were then blocked with goat serum and incubated with anti-rat COX IV antibodies (sc–58348, Santa Cruz Biotechnology, Inc.). Immunoreactivity was visualized using an ABC staining system (Vector Laboratories, Burlington, CA, USA), according to the manufacturer’s instructions. The sections were counterstained with Mayer’s hematoxylin.

### 2.13. Statistical Analysis

Data were expressed as mean ± SD or number (percentage). Each cell experiment was performed in triplicate. In the animal experiments, each group contained six mice. The data were examined with a two-tailed t test for two independent groups. The significance level was set at *p* < 0.05. Statistical analysis was performed using SPSS version 25 for Windows (SPSS Inc., Chicago, IL, USA).

## 3. Results

### 3.1. Aldosterone Treatment Decreased Mitochondrial DNA and Protein in H9c2 Cells

**Figure 1:**
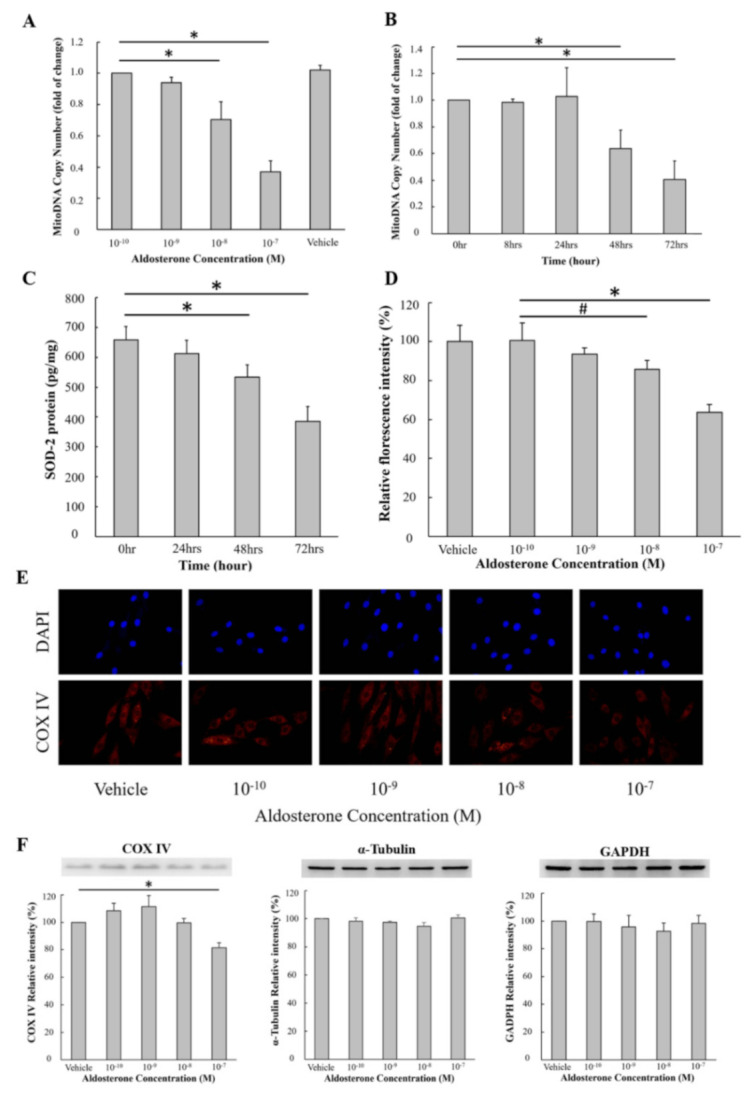
Aldosterone suppressed mitochondrial DNA and protein in a dose-dependent manner. (**A**) The effects of different dosages of aldosterone on H9c2 cells. H9c2 cells were treated with different concentrations of aldosterone (10^−10^, 10^−9^, 10^−8^ and 10^−7^ M) and vehicle (equal volume of DMSO) for 72 h. The mitochondrial DNA copy number was quantified by qPCR. (**B**) The effects of different durations of aldosterone treatment on H9c2 cells. H9c2 cells were treated with 10^−7^ M aldosterone and the mitochondrial copy number was quantified by qPCR at 0, 8, 24, 48 and 72 h. (**C**) The effects of different durations of aldosterone treatment on SOD2. H9c2 cells were treated with 10^−7^ M aldosterone, and the expression of SOD2 was determined by ELISA. (**D**) The dose effect of aldosterone on mitochondrial COX IV protein. H9c2 cells were treated with different concentrations of aldosterone (vehicle, 10^−10^, 10^−9^, 10^−8^ and 10^−7^ M) for 72 h. COX IV was stained with anti-COX IV antibodies. The fluorescence intensity of COX IV was measured using a fluorescence microscope. (**E**) H9c2 cells were treated with different concentrations of aldosterone (10^−10^, 10^−9^, 10^−8^ and 10^−7^ M) for 72 h. COX IV was stained with anti-COX IV antibodies (red), and nuclear DNA was stained with DAPI (blue). The representative images were captured using a fluorescence microscope; magnification ×400. (**F**) The effects of aldosterone on mitochondria (COX IV) and cytosolic (α-Tubulin and GAPDH) protein in H9c2 cells. H9c2 cells were treated with different concentrations of aldosterone (vehicle, 10^−10^, 10^−9^, 10^−8^ and 10^−7^ M) for 72 h. COX IV, α–Tubulin and GAPDH were determined using Western blot analysis. # *p* < 0.05 and * *p* < 0.01, compared between the two groups indicated by the line underneath.

H9c2 cells were treated with different concentrations of aldosterone (10^−10^, 10^−9^, 10^−8^ and 10^−7^ M) for 72 h. Mitochondrial DNA copy number significantly decreased after 72 h of treatment with 10^−8^ and 10^−7^ M aldosterone (Figure 1A). We then used 10^−7^ M aldosterone to examine the timing of this decrease in mitochondrial DNA copy number and found that the mitochondrial DNA copy number started to significantly decrease at 48 h and 72 h (Figure 1B). To evaluate the expression of the mitochondria-specific antioxidant enzyme SOD2, H9c2 cells were treated with different concentrations of aldosterone (10^−10^, 10^−9^, 10^−8^, and 10^−7^ M) for 72 h, and the concentration of SOD2 was measured using ELISA (Figure 1C). The results of ELISA showed that treatment with 10^−8^ and 10^−7^ M aldosterone resulted in significant decreases in SOD2 concentration (Figure 1C). We then stained the mitochondria-specific protein COX IV using different concentrations of aldosterone (vehicle, 10^−10^, 10^−9^, 10^−8^ and 10^−7^ M) in H9c2 cells for 72 h. The results showed that the fluorescence intensity of COX IV was significantly decreased after 72 h of treatment with 10^−8^ and 10^−7^ M aldosterone (Figure 1D,E). After 72 h of treatment with different concentrations of aldosterone (vehicle, 10^−10^ and 10^−7^ M), there was no significant difference in the amounts of annexin-V measured via flow cytometry and cytosolic cytochrome c measured via ELISA among different aldosterone concentrations (Figure S1A,B).

### 3.2. Aldosterone Treatment Mainly Decreased Mitochondria-Specific Protein but not Cytosolic Protein

H9c2 cells were treated with different concentrations of aldosterone (10^−10^, 10^−9^, 10^−8^ and 10^−7^ M) for 72 h and the mitochondria-specific protein (COX IV) and cytosolic protein (GAPDH and α–Tubulin) were measured via Western blot. Aldosterone (10^−7^ M) only significantly decreased COX IV protein but not the GAPDH or α–Tubulin (Figure 1F).

### 3.3. Aldosterone Treatment Decreased Mitochondrial DNA and Protein in Mouse Primary Cardiomyocytes and Human AC16 Cardiomyocytes

Mouse primary cardiomyocytes were treated with different concentrations of aldosterone (vehicle, 10^−10^ and 10^−7^ M) for 72 h. Mitochondrial DNA copy number significantly decreased with 10^−7^ M aldosterone after 72 h of treatment (Figure S1C). We then stained the mitochondria-specific protein COX IV using different concentrations of aldosterone (vehicle, 10^−10^ and 10^−7^ M) in mouse primary cardiomyocytes for 72 h. The results showed that the fluorescence intensity of COX IV was significantly decreased after 72 h of treatment with 10^−7^ M aldosterone (Figure S1E).

Human AC16 cardiomyocytes were also treated with different concentrations of aldosterone (vehicle, 10^−10^ and 10^−7^ M) for 72 h. Mitochondrial DNA copy number significantly decreased with 10^−7^ M aldosterone after 72 h of treatment (Figure S1D). The mitochondria-specific protein COX IV was stained after we treated different concentrations of aldosterone (vehicle, 10^−10^ and 10^−7^ M) in human AC16 cardiomyocytes for 72 h. The results showed that the fluorescence intensity of COX IV was significantly decreased after 72 h of treatment with 10^−7^ M aldosterone (Figure S1F).

### 3.4. Aldosterone Treatment Decreased Mitochondrial DNA and Protein Via MR and MAPK/P38 Pathways

**Figure 2:**
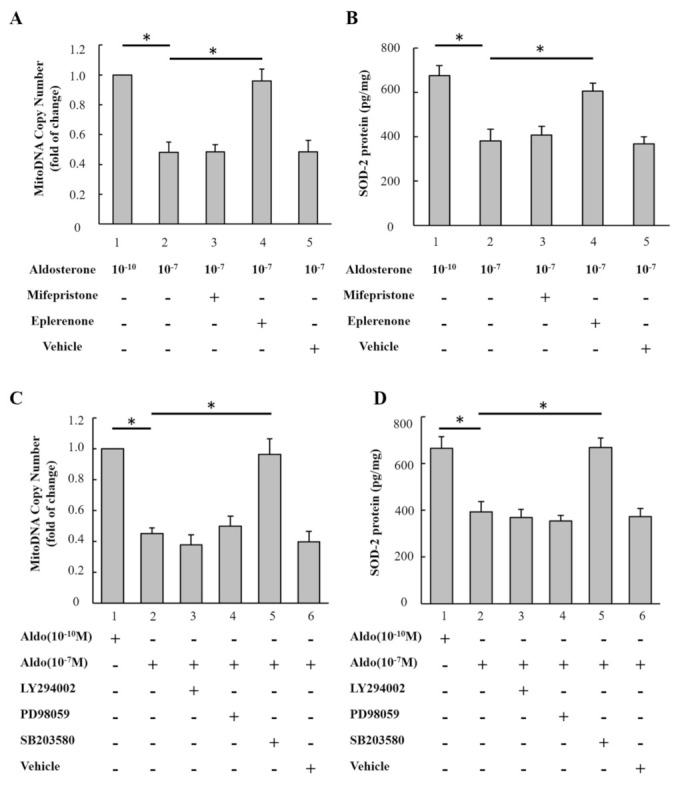
Aldosterone-associated reduction in mitochondrial DNA and protein via MR and MAPK/P38 pathway. (**A**,**B**) Role of MRs and glucocorticoid receptors on mitochondrial DNA and SOD2 protein. H9c2 cells were treated with 10^−7^ M eplerenone (an MR antagonist), 10^−7^ M mifepristone (a glucocorticoid receptor antagonist) and vehicle (equal volume of DMSO) for 1 h prior to 10^−7^ M aldosterone treatment. After 72 h, mitochondrial DNA was determined by qPCR and the expression of SOD2 was determined by ELISA. (**C**,**D**) Role of signaling mediators on mitochondrial DNA and SOD2. H9c2 cells were treated with 5 µg/mL SB203580 (an MAPK/p38 inhibitor), 50 µg/mL PD98059 (an MEK/ERK inhibitor), 50 µg/mL LY294002 (a PI3K/ AKT inhibitor) or vehicle (equal volume of DMSO) for 1 h prior to 10^−7^ M aldosterone treatment. After 72 h, mitochondrial DNA was determined by qPCR and the expression of SOD2 was determined by ELISA. * *p* < 0.01, compared between the two groups indicated by the line underneath.

We then investigated the mechanism underling aldosterone-induced mitochondrial dysfunction in H9c2 cells. Eplerenone (an MR blocker) significantly inhibited the effects of 10^−7^ M aldosterone on decreasing mitochondrial DNA copy number and SOD2 protein, but mifepristone (a glucocorticoid receptor blocker) did not. In addition, the effects of aldosterone on decreasing mitochondrial DNA copy number and SOD2 protein were inhibited by SB203580 (an MAPK/p38 inhibitor), but not by PD98059 (an MEK/ERK inhibitor) or LY294002 (a PI3K/ AKT inhibitor) (Figure 2A–D).

### 3.5. Aldosterone-Induced ROS Production in H9c2 Cells

**Figure 3:**
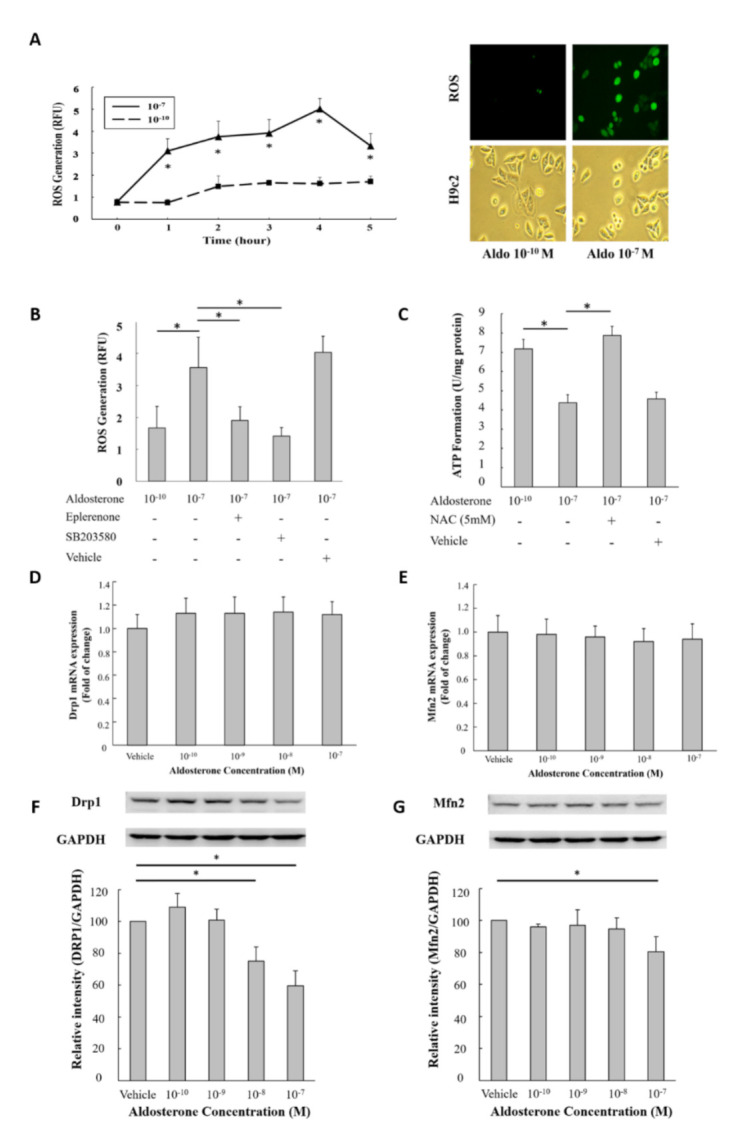
The MAPK/p38 signal pathway was associated with ROS production in aldosterone-treated H9c2 cells. (**A**) Aldosterone-induced ROS production in H9c2 cells. H9c2 cells were treated with 10^−7^ and 10^−10^ M aldosterone, and ROS regeneration was detected by reading the relative fluorescence from 0 to 5 h. The ROS fluorescence image of H9c2 cells treated with 10^−7^ and 10^−10^ M aldosterone at 45 min. The cell morphology under a light microscope was also examined; magnification ×400. (**B**) H9c2 cells were treated with 10^−7^ M eplerenone, 5 µg/mL SB203580 or vehicle (equal volume of DMSO) for 1 h prior to 10^−7^ M aldosterone treatment. After 1 h, ROS regeneration was detected by reading the relative fluorescence. (**C**) H9c2 cells were treated with 5 mM NAC (antioxidant, N–acetyl–L–cysteine) or vehicle (equal volume of ddH2O) for 1 h prior to 10^−7^ M aldosterone treatment. After 72 h, the ATP formation capability was determined using a Seahorse XF–24 extracellular flux analyzer. (**D**,**E**) The effects of aldosterone on fission (Drp1) and fusion (Mfn2) mRNA expression in H9c2 cells. H9c2 cells were treated with different concentrations of aldosterone (vehicle, 10^−10^, 10^−9^, 10^−8^ and 10^−7^ M) for 72 h. mRNA expression of Drp1 and Mfn2 were determined using quantitative RT-PCR. (**F**,**G**) The effects of aldosterone on fission (Drp1) and fusion (Mfn2) protein in H9c2 cells. H9c2 cells were treated with different concentrations of aldosterone (vehicle, 10^−10^, 10^−9^, 10^−8^ and 10^−7^ M) for 72 h. Drp1 and Mfn2 were determined using Western blot analysis. * *p* < 0.01 compared between the two groups indicated by the line underneath.

H9c2 cells were treated with 10^−10^ and 10^−7^ M aldosterone, and ROS generation was measured by reading the relative fluorescence. The results showed that ROS generation increased within 1 h after 10^−7^ M aldosterone treatment. In addition, the rate of ROS regeneration was significantly higher in the 10^−7^ M group compared with the 10^−10^ M group during 5 h of incubation (Figure 3A). Eplerenone (an MR blocker) and SB203580 (an MAPK/p38 inhibitor) significantly decreased ROS production (Figure 3B). Furthermore, the rate of mitochondrial ATP production as measured by Seahorse was significantly decreased after 10^−7^ M aldosterone treatment, and NAC significantly restored mitochondrial function (Figure 3C).

### 3.6. Aldosterone Decreased Both Mitochondrial Fusion and Fission Proteins but not Their mRNA Expression

Mitochondrial dynamics are also correlated with mitochondrial function in addition to the number of mitochondria. H9c2 cells were treated with different concentrations of aldosterone (10^−10^, 10^−9^, 10^−8^ and 10^−7^ M) for 72 h and the mitochondria fission (Drp1) and fusion (Mfn2) protein mRNA were measured via quantitative RT–PCR. There was no significant change of mRNA levels in H9c2 cells treated with different concentration of aldosterone (Figure 3D,E). There was a trend of the decreased fluorescence intensity of Drp1 after 72 h 10^−7^ M aldosterone treatment but not reaching statistical significance. The fluorescence intensity of Mfn2 remained stationary after 72 h of treatment with different concentrations of aldosterone (vehicle, 10^−10^, 10^−9^, 10^−8^ and 10^−7^ M) treatment (Figure S2A,B). The Drp1 and Mfn2 proteins were measured via Western blot in H9c2 cells treated with different concentrations of aldosterone (10^−10^, 10^−9^, 10^−8^ and 10^−7^ M). Both Drp1 and Mfn2 proteins were significantly decreased after 10^−7^ M aldosterone treatment (Figure 3E,F).

### 3.7. Aldosterone Decreased The Number of Mitochondria and Protein Expression in Cardiomyocytes Isolated from Aldosterone-Infused Mice

**Figure 4:**
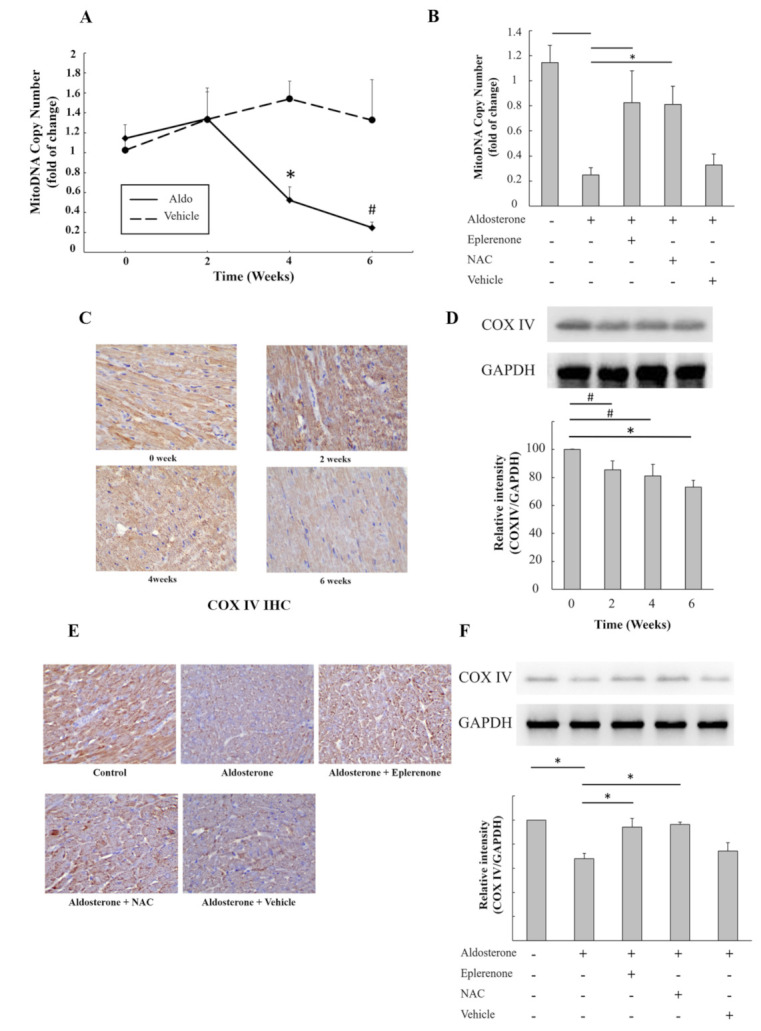
In vivo effect of aldosterone on mitochondrial DNA and COX IV protein in ICR mice. (**A**) Time course effect of aldosterone on heart tissue mitochondrial DNA in the aldosterone-infused ICR mice and controls. (**B**) Effect of an MR antagonist (eplerenone) and antioxidant (NAC, N–acetyl–L–cysteine) on cardiac mitochondrial DNA. (**C**) Immunohistochemical staining of cardiac mitochondrial COX IV protein after 0, 2, 4 and 6 weeks of aldosterone infusion; magnification ×400. (**D**) Western blot of cardiac COX IV protein and the corresponding quantitative results after 0, 2, 4 and 6 weeks of aldosterone infusion. (**E**) Immunohistochemical staining of cardiac mitochondrial COX IV protein after 6 weeks of aldosterone infusion in control and aldosterone-infused ICR mice treated with eplerenone, NAC and vehicle; magnification ×400. (**F**) Western blot of cardiac COX IV protein and the corresponding quantitative results after 6 weeks of aldosterone infusion in control and aldosterone-infused ICR mice treated with eplerenone, NAC and vehicle. # *p* < 0.05 and * *p* < 0.01, compared between the two groups indicated by the line underneath.

**Figure 5:**
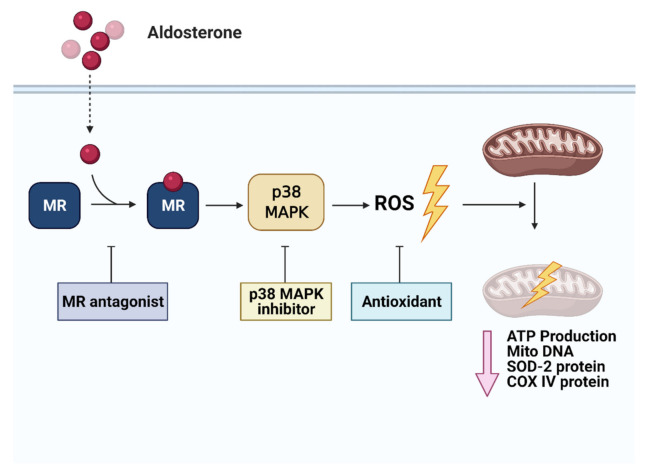
Schematic of the signaling of aldosterone-induced cardiac mitochondrial dysfunction in H9c2 cells. Aldosterone-induced cardiac mitochondrial dysfunction through MR/MAPK/p38 and ROS pathways. Mitochondrial DNA, SOD2, COX IV protein and ATP production were suppressed.

In aldosterone-infused ICR mice, the myocardial mitochondrial DNA copy number was measured using qPCR, and the results showed a significant decrease after 4 weeks of aldosterone infusion (Figure 4A). Both eplerenone and NAC treatment significantly reversed the decrease in mitochondrial DNA copy number induced by aldosterone infusion (Figure 4B). Immunohistochemical staining of mitochondrial COX IV protein showed a marked decrease in COX IV after 6 weeks of aldosterone infusion (Figure 4C). In addition, the expression of COX IV protein in heart tissue measured using Western blot was significantly reduced after 2 weeks of aldosterone infusion (Figure 4D). Both eplerenone and NAC treatment significantly reversed the decrease in COX IV protein induced by 6 weeks of aldosterone infusion (Figure 4E,F). The possible signaling pathway of aldosterone-induced cardiac mitochondrial dysfunction is summarized in Figure 5.

## 4. Discussion

In the present study, aldosterone significantly induced a decrease in mitochondria and mitochondrial dysfunction in vitro and in vivo, possibly through an MR/MAPK/p38 pathway and by inducing ROS generation. In addition, mitochondrial ATP production was suppressed after aldosterone treatment. These adverse effects of aldosterone on mitochondria may play a role in aldosterone-induced cardiac remodeling and dysfunction in various clinical conditions such as heart failure and PA.

Current management of heart failure focuses on energy-sparing strategies such as reductions in heart rate, preload and afterload to decrease cardiac workload [9], which play important roles in reducing myocardial oxygen consumption and maintaining a balance of energy supply and demand [13]. Mitochondrial dysfunction is also an important feature of heart failure, and mitochondrial function is a potential therapeutic target [13]. Mitochondria are intracellular double-membraned organelles that are major sources of energy production. Mitochondrial dysfunction will lead to organ failure, especially in organs with a high energy demand such as the brain and heart [17]. The heart is the most metabolically active organ, and mitochondria comprise about 25% of cell volume in human myocardium [18,19]. The rapid accumulation of mitochondrial DNA mutations was demonstrated to cause premature aging, cardiac remodeling, and heart failure in a mouse model [20]. In addition, the accumulation of mitochondrial DNA point mutations with age has been associated with decreased mitochondrial DNA content, and these mutations may result in target organ failure [20,21]. Moreover, a decrease in mitochondrial DNA has also been correlated with mitochondrial dysfunction. In this study, aldosterone, an important heart failure-related neuroendocrine hormone, significantly reduced mitochondrial DNA and mitochondrial ATP production in a dose-dependent manner.

Activation of the RAAS is a hallmark in the pathogenesis of heart failure and the progression of cardiac remodeling [22]. Aldosterone is the end product of the RAAS, and it plays a crucial role in electrolyte homeostasis, blood pressure regulation, and tissue remodeling [23]. The expression of aldosterone has been reported to increase in proportion to the severity of heart failure, and to be further increased after diuretic treatment [24]. In the Randomized Aldactone Evaluation Study (RALES) and Eplerenone Post myocardial infarction Heart failure Efficacy and SUrvival Study (EPHESUS), an aldosterone antagonist significantly improved cardiovascular outcomes [1,2]. Excess aldosterone has also been shown to induce cardiac remodeling and heart failure in PA patients [3], and PA patients have been shown to have worse cardiac hypertrophy and fibrosis independently of hemodynamic effects compared with essential hypertension patients [25]. ROS generation may contribute to the development of cardiac hypertrophy and fibrosis in patients with heart failure and PA [26], and increased oxidative stress has been associated with endothelial dysfunction, atherosclerosis, fibrosis and mitochondrial dysfunction [27]. In the present study, aldosterone induced ROS generation and resulted in a decrease in mitochondria and energy generation rate. This may contribute to chronic energy deficits and mechanical dysfunction of cardiomyocytes in heart failure and PA.

Recent studies have suggested that mitochondrial dysfunction and imbalanced mitochondrial dynamics play important roles in cardiac remodeling and the development of heart failure. Ibarrola et al. reported that aldosterone induced oxidative stress and decreased the expression of peroxisome proliferator-activated receptor gamma coactivator 1–alpha (PGC–1α) and the production of mitochondrial DNA in human cardiac fibroblasts. Mitochondrial DNA and PGC–1α have also been shown to be suppressed in patients with aortic stenosis [28]. However, we used cardiomyocytes in the present study unlike Ibarrola’s study, which could provide information on the direct effect of aldosterone on cardiac structure and function. In addition, we found that the molecular pathway for the effect of aldosterone on mitochondria was through MR and MAPK and p38 activation.

In the current studies, we first demonstrated that aldosterone activated the MR and the common downstream second messengers involving the MR signaling were examined including PI3K/Akt, MAPK/p38 and MEK/ERK. These pathways involved in both genomic and non-genomic pathways of aldosterone and MR [29]. Only the MAPK/p38 inhibitor could restore the aldosterone-induced mitochondria dysfunction in H9c2 cells. MAPK and p38 play crucial roles in ROS generation and are associated with cardiomyopathy [30]. Kumphun et al. also demonstrated that p38 MAPK inhibition could protect mitochondrial function after ischemia and reperfusion injury [31]. In the present study, p38 MAPK inhibition almost completely abolished the effects of aldosterone on ROS generation and mitochondrial DNA and SOD2 protein suppression. In addition, the ROS increased within 1 h after aldosterone treatment which implies that these effects were through both genomic and non-genomic pathways. Previous studies showed that the rapid non-genomic phenomena are guided by three different pathways involving MR activation or by cross-talk, via c–Src activation, with other receptors such as angiotensin II receptor type 1 (AT1R), estrogen receptor (GEPR) and epidermal growth factor receptor (EGFR) [29]. Both genomic and non-genomic pathways contributed to the inflammation and fibrosis. A previous study showed that ERK was involved in the non-genomic pathway of aldosterone-induced cardiac inflammation and fibrosis [32]. However, the ERK inhibitor could not abolish the detrimental effects of aldosterone to cardiomyocytes in current study. Further studies are needed to examine the role of the non-genomic pathway and its interplay with the genomic pathway in the aldosterone-induced mitochondria dysfunction.

In the normal physiological condition, corticosteroid and aldosterone shared equally affinity to the MR but the serum concertation of corticosteroid was much higher. For that reason, most MRs were occupied by the corticosteroid. 11β–Hydroxysteroid dehydrogenase type 2 (11β–HSD2) inactivated glucocorticoids and restored the MR selectivity for aldosterone. However, the activity of 11β–HSD2 was low in the cardiomyocytes [33] and the role of corticosteroid on MR remained unclear. In our in vivo model, aldosterone infusion mice still presented with cardiac mitochondria dysfunction, as previous studies demonstrated [29]. The possible mechanisms of selective MR activation by aldosterone including ligand-sensitive transactivation, subcellular compartmentalization or posttranslational modifications of MR. In addition, these mechanisms might be altered in pathophysiological situations [34,35]. The role of GR was also important since each steroid could bind both receptors. Our previous study showed aldosterone increased the tissue inhibitor of metalloproteinases–1 (TIMP–1) expression via GR resulted in enhanced collagen accumulation via the suppression of matrix metalloproteinase–1 activity in cardiac fibroblasts [36]. However, in the current study, the GR blocker, mifepristone could not abolish the effect of aldosterone which suggest that the GR was not involved in the pathway.

Mitochondria are highly dynamic organelles that undergo coordinated cycles of fission and fusion, and the balance of mitochondrial fusion and fission is necessary to support mitochondrial function. Interestingly, the mitochondria fission protein, Drp1, and fusion protein, Mfn–2, were both decreased in H9c2 cells after aldosterone treatment but the mRNA expression of Drp1 and Mfn2 remained stationary. Shen et al. demonstrated that total Drp1 expression was not suppressed after TNF–α treatment, but that mitochondrial Drp1 and phosphorated Drp1 were upregulated [37]. In addition, the phosphorylation status of Drp1 was not a determinant of Drp1 recruitment to mitochondria. In the current study, it seems that the fission and fusion proteins are not involved in the aldosterone-induced mitochondria dysfunction. However, the detailed mechanism of mitochondrial dynamics involved in aldosterone-induced cardiac mitochondrial dysfunction remains elusive, and further studies are required to explore this issue [38].

There are several limitations to the current study. First, the major findings were based on cell and mouse studies, and whether they occur in clinical conditions is not known. However, the present study provides good evidence to support the potential mechanisms of aldosterone-induced cardiac mitochondrial dysfunction in heart failure and PA patients. Second, many other factors such as increased inflammation, sympathetic system activation and elevated angiotensin II are associated with heart failure and may interfere with the effects of aldosterone on cardiac mitochondria. However, these factors were not explored in this study.

## 5. Conclusions

In conclusion, we demonstrated that aldosterone excess induced a decrease in mitochondria and mitochondrial dysfunction via MRs and oxidative stress in vitro and in vivo. There were three major findings. First, aldosterone downregulated mitochondrial DNA, SOD2 and COX IV protein expressions and mitochondrial function as assessed by ATP production. Second, aldosterone suppressed mitochondria through an MR/MAPK/p38 pathway and ROS production. Third, we demonstrated a corresponding phenomenon in an ICR mouse model. We also proposed the mechanisms of aldosterone-induced cardiac mitochondrial dysfunction, which may provide potential therapeutic targets for aldosterone-induced cardiac remodeling and dysfunction.

## Data Availability

The original contributions presented in the study are included in the article. Further inquiries can be directed to the corresponding authors.

## Supplementary Materials

The following are available online at https://www.mdpi.com/article/10.3390/biomedicines9080946/s1, Figure S1: Effects of aldosterone on H9c2 cells, mouse primary cardiomyocytes and human AC16 cardiomyocytes, Figure S2: The effects of aldosterone on mitochondria fission and fusion protein.

## References

[1] Pitt B., Zannad F., Remme W.J., Cody R., Castaigne A., Perez A., Palensky J., Wittes J. (1999). The effect of spironolactone on morbidity and mortality in patients with severe heart failure. Randomized Aldactone Evaluation Study Investigators. N. Engl. J. Med..

[2] Pitt B., Remme W., Zannad F., Neaton J., Martinez F., Roniker B., Bittman R., Hurley S., Kleiman J., Gatlin M. (2003). Eplerenone, a selective aldosterone blocker, in patients with left ventricular dysfunction after myocardial infarction. N. Engl. J. Med..

[3] Tsai C.-H., Pan C.-T., Chang Y.-Y., Chen Z.-W., Wu V.-C., Hung C.-S., Lin Y.-H. (2021). Left ventricular remodeling and dysfunction in primary aldosteronism. J. Hum. Hypertens..

[4] Chen Z.-W., Tsai C.-H., Pan C.-T., Chou C.-H., Liao S.-C., Hung C.-S., Wu V.-C., Lin Y.-H. (2019). TAIPAI Study Group Endothelial Dysfunction in Primary Aldosteronism. Int. J. Mol. Sci..

[5] Sun Y., Zhang J., Lu L., Chen S.S., Quinn M.T., Weber K.T. (2002). Aldosterone-induced inflammation in the rat heart: Role of oxidative stress. Am. J. Pathol..

[6] Wu V.-C., Wang S.-M., Chang C.-H., Hu Y.-H., Lin L.-Y., Lin Y.-H., Chueh S.-C.J., Chen L., Wu K.-D. (2016). Long term outcome of Aldosteronism after target treatments. Sci. Rep..

[7] Hock M.B., Kralli A. (2009). Transcriptional control of mitochondrial biogenesis and function. Annu. Rev. Physiol..

[8] Zhao Q., Sun Q., Zhou L., Liu K., Jiao K. (2019). Complex Regulation of Mitochondrial Function During Cardiac Development. J. Am. Hear. Assoc..

[9] Sabbah H.N. (2020). Targeting the Mitochondria in Heart Failure: A Translational Perspective. JACC Basic Transl. Sci..

[10] Korge P., Ping P., Weiss J.N. (2008). Reactive oxygen species production in energized cardiac mitochondria during hypoxia/reoxygenation: Modulation by nitric oxide. Circ. Res..

[11] Rosca M.G., Tandler B., Hoppel C.L. (2013). Mitochondria in cardiac hypertrophy and heart failure. J. Mol. Cell. Cardiol..

[12] Murphy S.P., Kakkar R., McCarthy C.P., Januzzi J.L. (2020). Inflammation in Heart Failure: JACC State-of-the-Art Review. J. Am. Coll. Cardiol..

[13] Brown D.A., Perry J.B., Allen M.E., Sabbah H.N., Stauffer B.L., Shaikh S.R., Cleland J.G., Colucci W.S., Butler J., Voors A.A. (2017). Expert consensus document: Mitochondrial function as a therapeutic target in heart failure. Nat. Rev. Cardiol..

[14] Li D., Wu J., Bai Y., Zhao X., Liu L. (2014). Isolation and culture of adult mouse cardiomyocytes for cell signaling and in vitro cardiac hypertrophy. J. Vis. Exp..

[15] Jeong S.H., Kim H.K., Song I.S., Noh S.J., Marquez J., Ko K.S., Rhee B.D., Kim N., Mishchenko N.P., Fedoreyev S.A. (2014). Echinochrome a increases mitochondrial mass and function by modulating mitochondrial biogenesis regulatory genes. Mar. Drugs.

[16] Chung E., Joiner H.E., Skelton T., Looten K.D., Manczak M., Reddy P.H. (2017). Maternal exercise upregulates mitochondrial gene expression and increases enzyme activity of fetal mouse hearts. Physiol. Rep..

[17] Hsu Y.-H.R., Yogasundaram H., Parajuli N., Valtuille L., Sergi C., Oudit G.Y. (2015). MELAS syndrome and cardiomyopathy: Linking mitochondrial function to heart failure pathogenesis. Hear. Fail. Rev..

[18] Schaper J., Meiser E., Stämmler G. (1985). Ultrastructural morphometric analysis of myocardium from dogs, rats, hamsters, mice, and from human hearts. Circ. Res..

[19] Barth E., Stammler G., Speiser B., Schaper J. (1992). Ultrastructural quantitation of mitochondria and myofilaments in cardiac muscle from 10 different animal species including man. J Mol. Cell. Cardiol..

[20] Kraytsberg Y., Nekhaeva E., Bodyak N.B., Khrapko K. (2003). Mutation and intracellular clonal expansion of mitochondrial genomes: Two synergistic components of the aging process?. Mech. Ageing Dev..

[21] Simmons R.A., Suponitsky-Kroyter I., Selak M.A. (2005). Progressive accumulation of mitochondrial DNA mutations and decline in mitochondrial function lead to beta-cell failure. J. Biol. Chem..

[22] Hartupee J., Mann D.L. (2017). Neurohormonal activation in heart failure with reduced ejection fraction. Nat. Rev. Cardiol..

[23] Funder J.W. (2017). Aldosterone and Mineralocorticoid Receptors—Physiology and Pathophysiology. Int. J. Mol. Sci..

[24] Zannad F. (1995). Aldosterone and heart failure. Eur. Heart J..

[25] Rossi G.P., Di Bello V., Ganzaroli C., Sacchetto A., Cesari M., Bertini A., Giorgi D., Scognamiglio R., Mariani M., Pessina A.C. (2002). Excess ldosterone is Associated With Alterations of Myocardial Texture in Primary Aldosteronism. Hypertension.

[26] Takimoto E., Kass D.A. (2007). Role of oxidative stress in cardiac hypertrophy and remodeling. Hypertension.

[27] Panth N., Paudel K.R., Parajuli K. (2016). Reactive Oxygen Species: A Key Hallmark of Cardiovascular Disease. Adv. Med..

[28] Ibarrola J.F., Sadaba R., Martinez-Martinez E., Garcia-Peña A., Arrieta V., Alvarez V., Fernández-Celis A., Gainza A., Cachofeiro V., Santamaria E. (2018). Aldosterone Impairs Mitochondrial Function in Human Cardiac Fibroblasts via A-Kinase Anchor Protein 12. Sci. Rep..

[29] Cannavo A., Bencivenga L., Liccardo D., Elia A., Marzano F., Gambino G., D’Amico M.L., Perna C., Ferrara N., Rengo G. (2018). Aldosterone and Mineralocorticoid Receptor System in Cardiovascular Physiology and Pathophysiology. Oxidative Med. Cell. Longev..

[30] Wang S., Ding L., Ji H., Xu Z., Liu Q., Zheng Y. (2016). The Role of p38 MAPK in the Development of Diabetic Cardiomyopathy. Int. J. Mol. Sci..

[31] Kumphune S., Surinkaew S., Chattipakorn S.C., Chattipakorn N. (2015). Inhibition of p38 MAPK activation protects cardiac mitochondria from ischemia/reperfusion injury. Pharm. Biol..

[32] Cannavo A., Liccardo D., Eguchi A., Elliott K.J., Traynham C.J., Ibetti J., Eguchi S., Leosco D., Ferrara N., Rengo G. (2016). Myocardial pathology induced by aldosterone is dependent on non-canonical activities of G protein-coupled receptor kinases. Nat. Commun..

[33] Lombes M., Alfaidy N., Eugene E., Lessana A., Farman N., Bonvalet J.P. (1995). Prerequisite for cardiac aldosterone action. Mineralocorticoid receptor and 11 beta-hydroxysteroid dehydrogenase in the human heart. Circulation.

[34] Farman N., Rafestin-Oblin M.E. (2001). Multiple aspects of mineralocorticoid selectivity. Am. J. Physiol. Renal Physiol..

[35] Muller O., Pradervand S., Berger S., Centeno G., Milet A., Nicod P., Pedrazzini T., Tronche F., Schütz G., Chien K. (2007). Identification of corticosteroid-regulated genes in cardiomyocytes by serial analysis of gene expression. Genomics.

[36] Hung C.S., Chou C.H., Liao C.W., Lin Y.T., Wu X.M., Chang Y.Y., Chen Y.H., Wu V.C., Su M.J., Ho Y.L. (2016). Aldosterone Induces Tissue Inhibitor of Metalloproteinases-1 Expression and Further Contributes to Collagen Accumulation: From Clinical to Bench Studies. Hypertension.

[37] Shen Y., Shi Y., Chen G., Wang L., Zheng M., Jin H., Chen Y.-Y. (2018). TNF-α induces Drp1-mediated mitochondrial fragmentation during inflammatory cardiomyocyte injury. Int. J. Mol. Med..

[38] Yu R., Liu T., Ning C., Tan F., Jin S.-B., Lendahl U., Zhao J., Nistér M. (2019). The phosphorylation status of Ser-637 in dynamin-related protein 1 (Drp1) does not determine Drp1 recruitment to mitochondria. J. Biol. Chem..

