# Supplementary material for: Aldosterone Excess Induced Mitochondria Decrease and Dysfunction via Mineralocorticoid Receptor and Oxidative Stress In Vitro and In Vivo
Source: Biomedicines. 2021 Aug 2;9(8):946. doi: 10.3390/biomedicines9080946 (PMC8392669; doi:10.3390/biomedicines9080946)
Supplement: Supplementary file 1 [file biomedicines-09-00946-s001.zip › biomedicines-1328882 - supplementary materials.pdf]

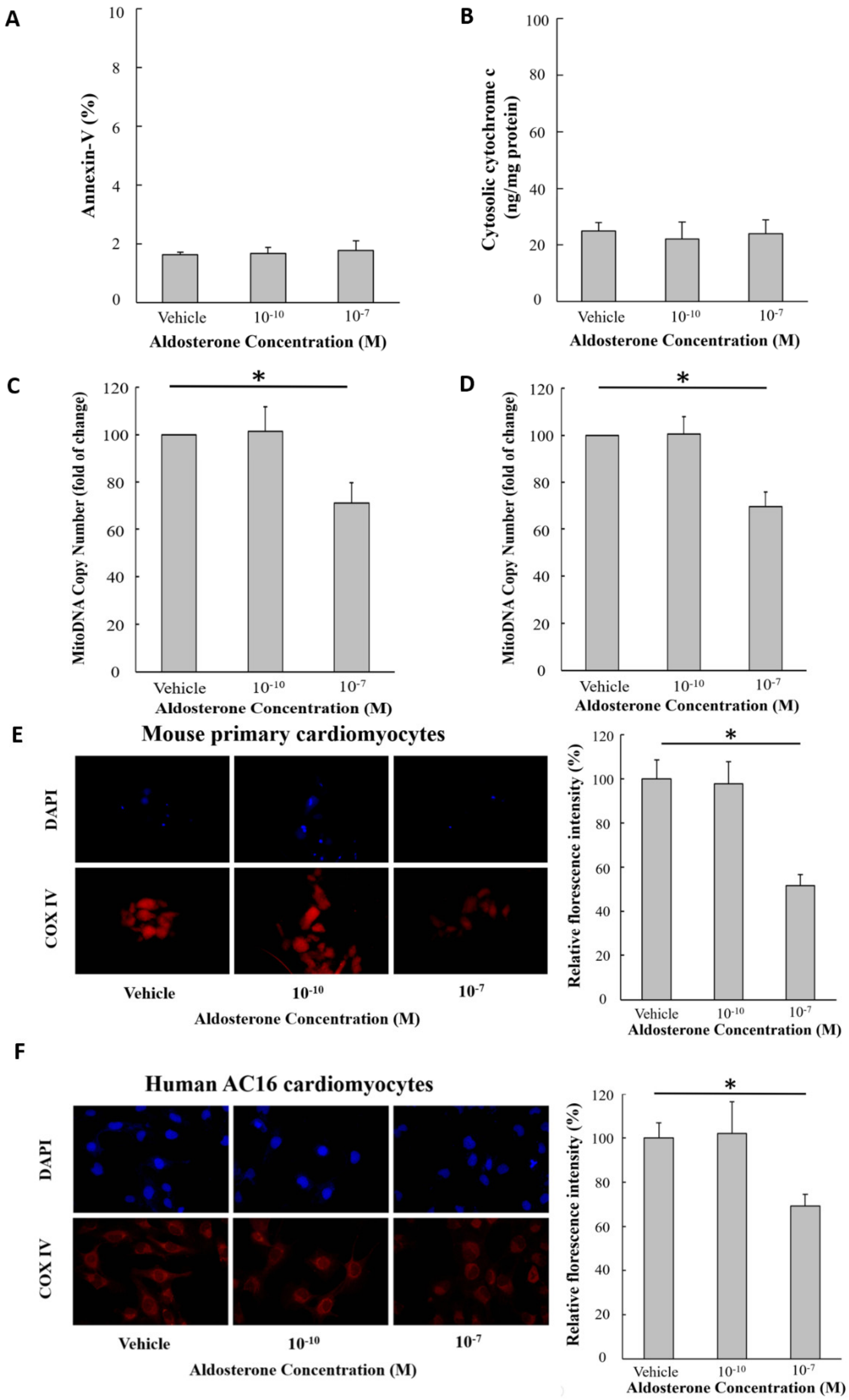

Figure S1. Effects of aldosterone on H9c2 cells, mouse primary cardiomyocytes and human AC16 cardiomyocytes. (**A**) Percentage of annexin-V expression in H9c2 cells treated with different concentrations of aldosterone (10^{-10}, 10^{-7} M) and vehicle (equal volume of DMSO) for 72 h measured by flow cytometry analysis. (**B**) Percentage of cytosolic cytochrome c expression in H9c2 cells treated with different concentrations of aldosterone (10^{-10}, 10^{-7} M) and vehicle (equal volume of DMSO) for 72 h which was determined by ELISA. (**C**) The dose effect of aldosterone on mitochondrial DNA in mouse primary cardiomyocytes. Mouse primary cardiomyocytes with different concentrations of aldosterone (10^{-10}, 10^{-7} M) and vehicle (equal volume of DMSO) for 72 h. The mitochondrial DNA copy number was quantified by qPCR. (**D**) The dose effect of aldosterone on mitochondrial DNA in human A16 cardiomyocytes. Human A16 cardiomyocytes with different concentrations of aldosterone (10^{-10}, 10^{-7} M) and vehicle (equal volume of DMSO) for 72 h. The

mitochondrial DNA copy number was quantified by qPCR. (**E**) The dose effect of aldosterone on mitochondrial COX IV protein in mouse primary cardiomyocytes. Mouse primary cardiomyocytes with different concentrations of aldosterone (10^{-10}, 10^{-7} M) and vehicle (equal volume of DMSO) for 72 h. COX IV was stained with anti-COX IV antibodies (red), and nuclear DNA was stained with DAPI (blue). The fluorescence intensity of COX IV was measured using a fluorescence microscope; magnification ×400. (**F**) The dose effect of aldosterone on mitochondrial COX IV protein in human AC16 cardiomyocytes. human AC16 cardiomyocytes with different concentrations of aldosterone (10^{-10}, 10^{-7} M) and vehicle (equal volume of DMSO) for 72 h. COX IV was stained with anti-COX IV antibodies (red), and nuclear DNA was stained with DAPI (blue). The fluorescence intensity of COX IV was measured using a fluorescence microscope; magnification ×400.

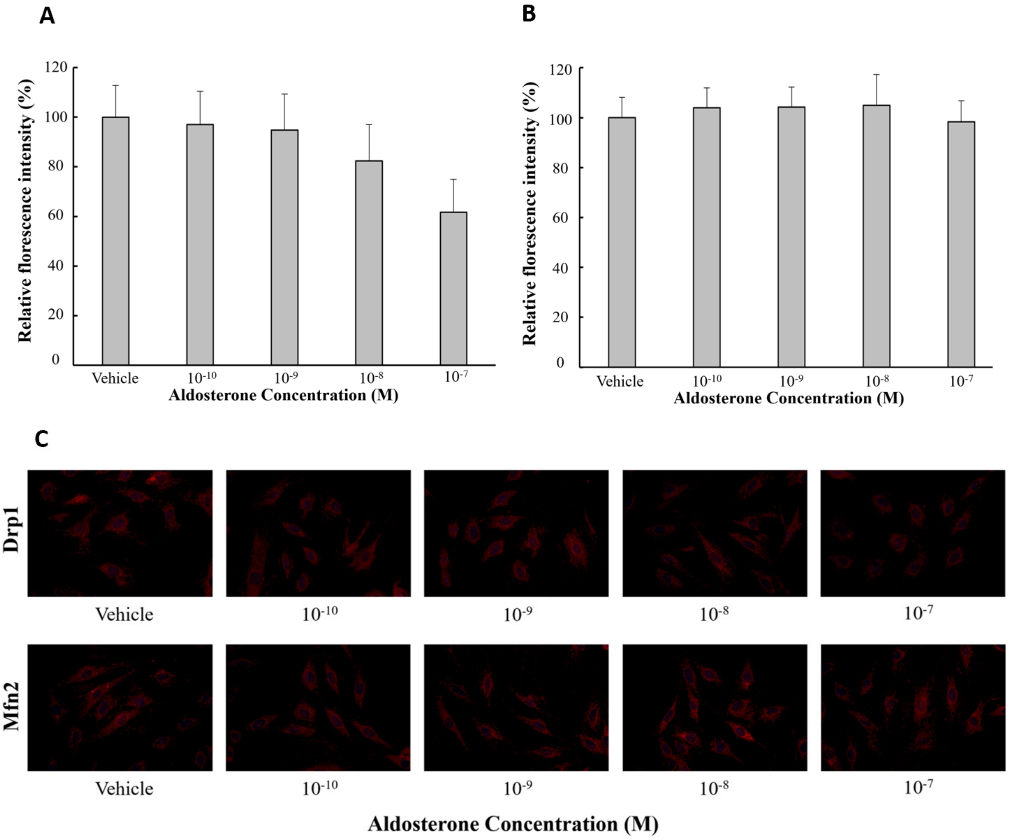

Figure S2. The effects of aldosterone on mitochondria fission and fusion protein. (**A**) The effects of aldosterone on fission (Drp1) protein in H9c2 cells. H9c2 cells were treated with different concentrations of aldosterone (vehicle, 10^{-10}, 10^{-9}, 10^{-8}, and 10^{-7} M) for 72 h. Drp1 were determined using fluorescence intensity. (**B**) The effects of aldosterone on fusion (Mfn2) protein in H9c2 cells. H9c2 cells were treated with different concentrations of aldosterone (vehicle, 10^{-10}, 10^{-9}, 10^{-8}, and 10^{-7} M) for 72 h. Mfn2 were determined using fluorescence intensity. (**C**) Drp1 and Mfn2 were stained with anti-Drp1 and anti-Mfn2 antibodies (red), respectively. The fluorescence intensity of COX IV was measured using a fluorescence microscope; magnification ×400.
